# Chronic splenic artery occlusion with gastric wall arterial ectasia, an unusual cause of massive upper gastrointestinal hemorrhage

**DOI:** 10.1259/bjrcr.20200194

**Published:** 2021-02-24

**Authors:** Hassan Al-Balas, Zeyad A. Metwalli, David M. Sada

**Affiliations:** 1 Michael E. DeBakey VA Medical Center, Houston, Texas, USA; 2 Jordan University of Science and Technology, Irbid, Jordan; 3 Baylor College of Medicine, One Baylor Plaza, Houston, Texas, USA; 4 University of Texas M.D. Anderson Cancer Center, Houston, Texas, USA

## Abstract

Life-threatening upper gastrointestinal (GI) hemorrhage can occur as a result of bleeding from a variety of arterial and venous sources. We present an unusual cause of life-threatening upper GI hemorrhage arising from ectatic gastric wall arterial branches in a 49-year-old male with previously unrecognized chronic splenic artery thrombosis. The patient developed a recurrence of bleeding despite coil embolization of an accessory left gastric artery branch supplying the gastric fundus suspected to be the site of active bleeding. The patient subsequently underwent splenectomy and surgical ligation of a bleeding gastric artery branch. This case emphasizes the importance of recognizing this unusual cause of upper GI hemorrhage for proper management and prevention of recurrence. Informed consent was obtained from the patient for publication of the case report including accompanying images.

## Clinical presentation

A 49-year-old male with history of poorly controlled Type 2 diabetes was admitted with chronic lower extremity ischemia complicated by necrotizing fasciitis. He underwent surgical below-knee amputation. On the seventh post-operative day, the patient experienced hypotension and massive hematemesis. He was then transferred to the critical care unit for resuscitation and blood transfusion. A CT angiogram was obtained, which showed active extravasation arising from a peripheral branch in the gastric fundus.

## Investigation and management

**Figure 1.**
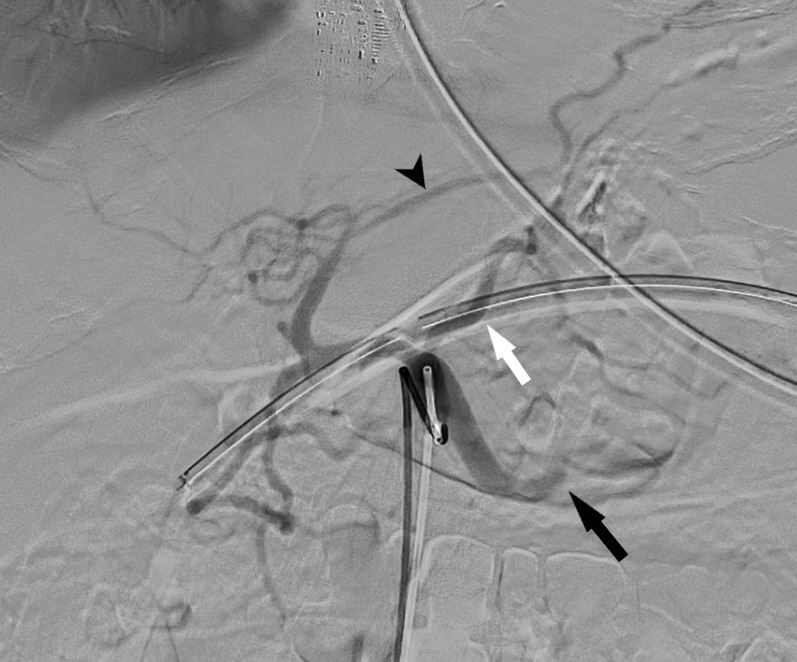
Celiac angiogram showing occlusion of the mid-splenic artery (black arrow) with prominent left gastric (white arrow) and accessory left gastric (black arrowhead) arteries.

**Figure 2.**
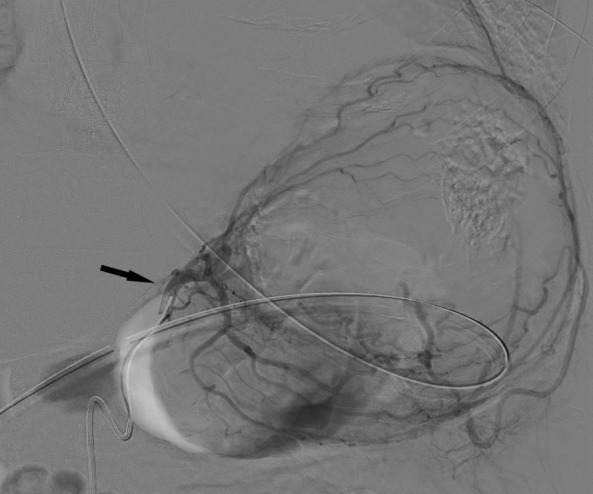
Selective left gastric (black arrow) angiogram shows prominent gastric wall vessels with collateral blood supply to the spleen.

**Figure 3.**
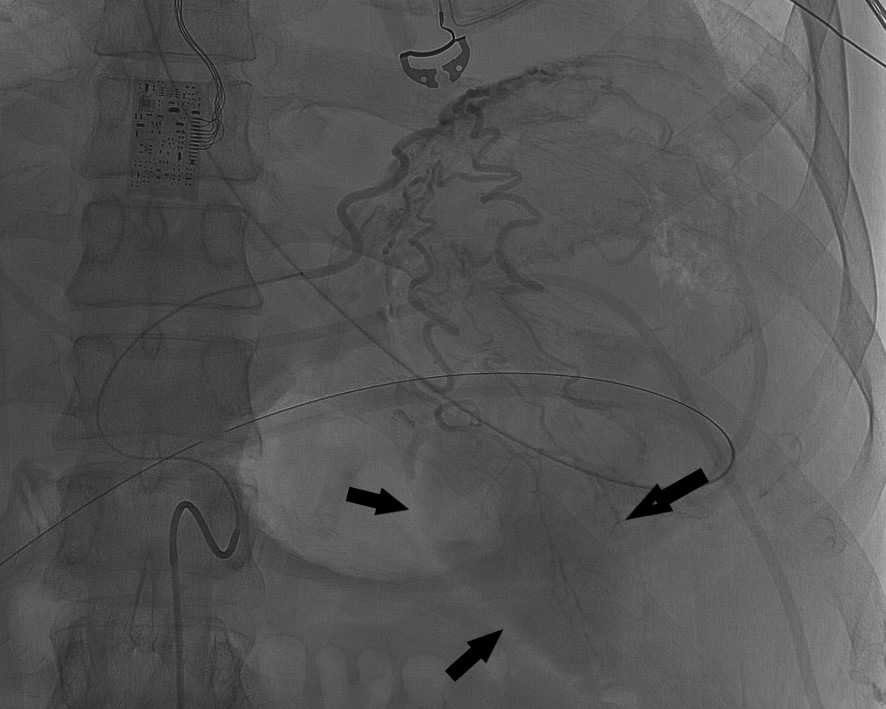
Selective accessory left gastric artery angiogram arising from left hepatic artery shows prominent gastric wall collaterals with delayed opacification of splenic parenchyma (black arrows).

**Figure 4.**
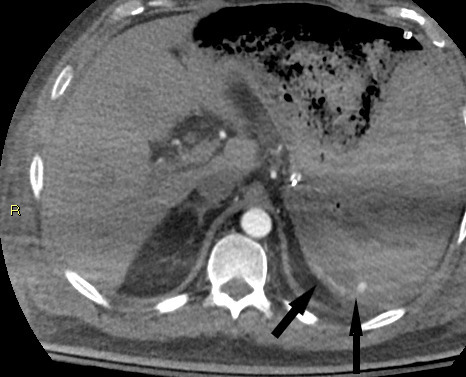
Contrast-enhanced CT of the upper abdomen shows active contrast extravasation (black arrows) from the fundal gastric wall consistent with active bleeding.

Emergency upper endoscopy showed a large amount of blood within the gastric lumen limiting full gastric evaluation. No active hemorrhage was identifiable at that time. Endoscopy also showed prominent submucosal variceal-like structures in the gastric fundus and along the body of the stomach. No esophageal varices were seen. The patient was not known to have chronic liver disease or portal hypertension. He developed recurrent bleeding the following morning and a mesenteric angiogram was performed. Celiac angiography (Figure 1) showed chronic occlusion of the mid-splenic artery and an enlarged left gastric artery as well as a prominent accessory left gastric artery arising from the left hepatic artery. Selective angiograms of the left gastric artery (Figure 2) and accessory left gastric artery (Figure 3) showed ectatic peripheral gastric branches with retrograde opacification of short gastric artery branches. These branches supplied splenic parenchyma, which was enhanced on delayed phase imaging. No active contrast extravasation was seen. Therefore, empiric embolization of the distal accessory left gastric artery supplying the gastric fundus at the site of previously documented extravasation on CT was performed (Figure 4). This CT also demonstrated splenic artery occlusion, possibly related to atherosclerosis in this patient with diabetes and peripheral vascular disease. Embolization was performed using platinum fibered coils.

2 days later, the patient developed recurrent hematemesis and underwent emergency laparotomy with ligation of a bleeding gastric vessel and splenectomy. The patient was discharged home after few days without recurrence of upper GI hemorrhage on 18 month follow-up.

## Discussion

Peptic ulcer disease, esophageal varices and gastritis remain the leading causes of upper gastrointestinal hemorrhage with an overall mortality rate of 6–10%.^1,2^ Upper gastrointestinal hemorrhage is usually managed by supportive care and transfusion therapy followed by endoscopy as needed.^3^ In the clinical case presented, the endoscopic finding of prominent submucosal vessels in the absence of stigmata of portal hypertension or history of cirrhosis was not easily explainable without further investigation.

The subsequent celiac angiogram showed chronic thrombosis of the mid-splenic artery with prominent left gastric and accessory left gastric arteries with enlarged gastric wall branches. Both arteries supplied the spleen via enlarged arterial collateral vessels within the gastric wall with reversed flow within the short gastric arteries, a known collateral arterial pathway to the spleen.^4^ This explains the unusual finding of prominent submucosal gastric vessels seen at endoscopy. Possible etiologies of splenic artery thrombosis include atherosclerosis, trauma, thrombosed aneurysm and pancreatitis.^5^ The etiology of splenic artery thrombosis in the presented case is unknown and was asymptomatic prior to presentation of life-threatening upper gastrointestinal hemorrhage. In this patient with severe peripheral vascular disease, splenic artery occlusion likely occurred gradually allowing the formation of a robust collateral pathway, while preserving perfusion to the spleen in its entirety.

Few case reports described similar cases of upper gastrointestinal hemorrhage secondary to enlarged gastric wall arterial collaterals as a result of chronic splenic artery occlusion secondary to various etiologies including congenital and acquired causes.^6–8^ However, our case is unique as the gastric wall collaterals were supplied by an enlarged accessory left gastric artery arising from left hepatic artery as well as left gastric artery.

To prevent recurrent bleeding from gastric wall arterial collaterals, splenectomy was performed. By performing splenectomy, the underlying driving etiology of reversal of blood flow in the short gastric arteries and increased blood flow within these collateral vessels was addressed, therefore effectively preventing recurrence of upper gastrointestinal hemorrhage.^8^

## Learning points

1. Chronic splenic artery occlusion should be recognized as a rare cause of upper gastrointestinal hemorrhage.
2. Patients with congenital or acquired splenic artery occlusion may be at increased risk of upper gastrointestinal hemorrhage and clinicians should consider flow-related changes through collateral pathways as a potential cause of bleeding in these patients.
3. Angiographers should be familiar with collateral circulation pathways to the spleen and its anatomic variations.
4. Splenectomy is a potential definitive therapy for patients presenting with upper gastrointestinal hemorrhage secondary to chronic splenic artery occlusion.
